# *Candida albicans* Heat Shock Proteins and Hsps-Associated Signaling Pathways as Potential Antifungal Targets

**DOI:** 10.3389/fcimb.2017.00520

**Published:** 2017-12-19

**Authors:** Ying Gong, Tao Li, Cuixiang Yu, Shujuan Sun

**Affiliations:** ^1^School of Pharmaceutical Sciences, Shandong University, Jinan, China; ^2^Intensive Care Unit, Qianfoshan Hospital Affiliated to Shandong University, Jinan, China; ^3^Respiration Medicine, Qianfoshan Hospital Affiliated to Shandong University, Jinan, China; ^4^Department of Pharmacy, Qianfoshan Hospital Affiliated to Shandong University, Jinan, China

**Keywords:** heat shock proteins, *Candida albicans*, signaling pathways, antifungal targets, virulence

## Abstract

In recent decades, the incidence of invasive fungal infections has increased notably. *Candida albicans (C. albicans)*, a common opportunistic fungal pathogen that dwells on human mucosal surfaces, can cause fungal infections, especially in immunocompromised and high-risk surgical patients. In addition, the wide use of antifungal agents has likely contributed to resistance of *C. albicans* to traditional antifungal drugs, increasing the difficulty of treatment. Thus, it is urgent to identify novel antifungal drugs to cope with *C. albicans* infections. Heat shock proteins (Hsps) exist in most organisms and are expressed in response to thermal stress. In *C. albicans*, Hsps control basic physiological activities or virulence via interaction with a variety of diverse regulators of cellular signaling pathways. Moreover, it has been demonstrated that Hsps confer drug resistance to *C. albicans*. Many studies have shown that disrupting the normal functions of *C. albicans* Hsps inhibits fungal growth or reverses the tolerance of *C. albicans* to traditional antifungal drugs. Here, we review known functions of the diverse Hsp family, Hsp-associated intracellular signaling pathways and potential antifungal targets based on these pathways in *C. albicans*. We hope this review will aid in revealing potential new roles of *C. albicans* Hsps in addition to canonical heat stress adaptions and provide more insight into identifying potential novel antifungal targets.

## Introduction

*C. albicans* causes superficial and potentially life-threatening systemic infections, especially in immunocompromised and high-risk surgical patients (Dimopoulos et al., 2007). Traditional antifungal drugs, such as azoles, polyenes, and echinocandins, mostly target *C. albicans*' cell envelopes, e.g., ergosterol on the plasma membrane or glucan on the cell wall (Odds et al., 2003). These antifungal drugs are extensively used in the clinic because of their high efficacy. However, traditional antifungal drugs have become increasingly ineffective against *C. albicans* infections due to multiple factors and resistance of *C. albicans* to these drugs has emerged more frequently (Kriengkauykiat et al., 2011; Wirk, 2011). Thus, it is urgent to develop novel antifungal agents based on identified potential targets in *C. albicans* (Wirk, 2011; Cuenca-Estrella, 2014).

**Figure 1:**
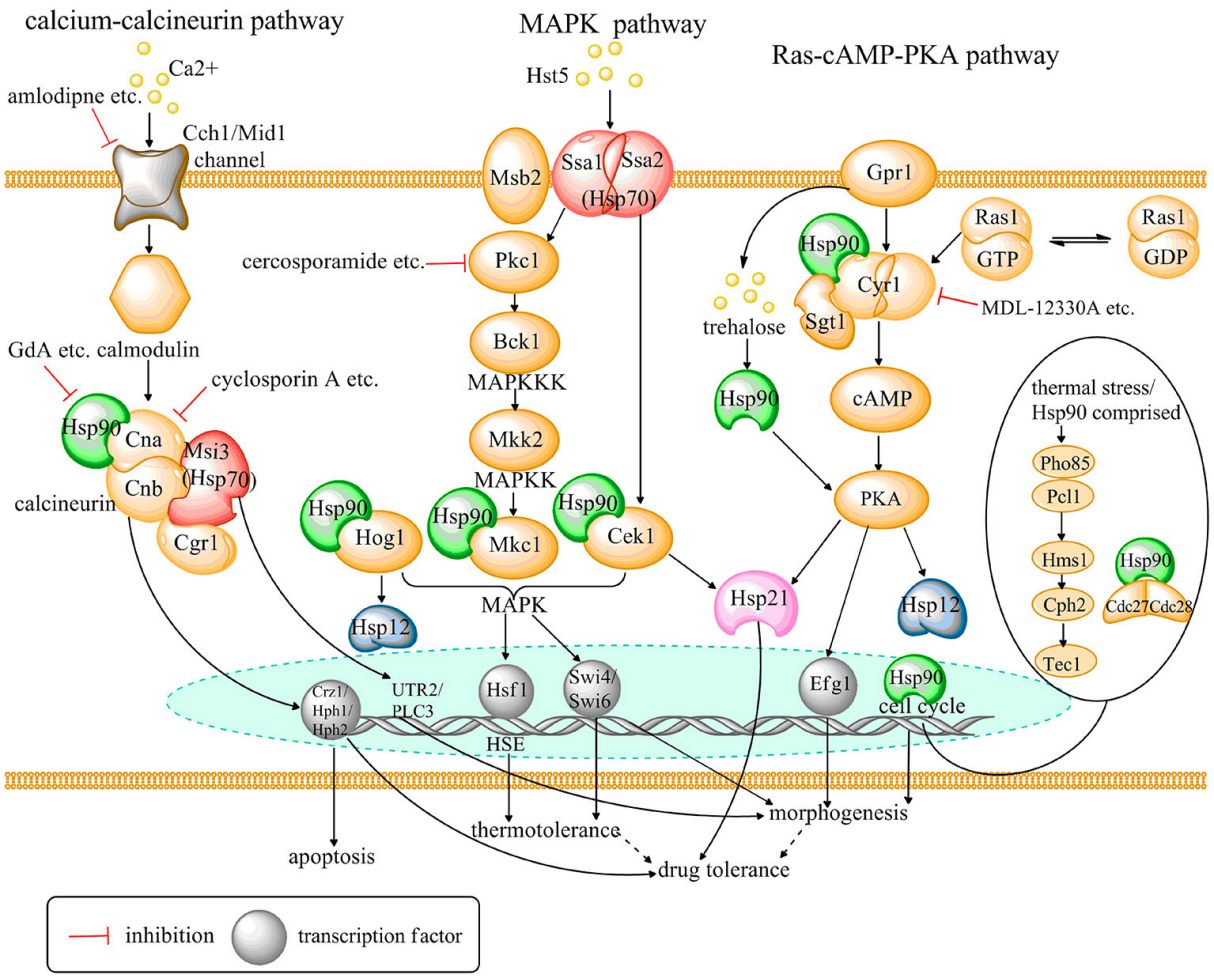
A schematic diagram depicting Hsp-associated signaling pathways and potential antifungal targets based on these pathways in *C. albicans*. Solid and dashed arrow lines indicate known pathways and putative pathways, respectively, which are predicted in the present study.

The Hsp family was first identified in *Drosophila melanogaster* in response to thermal stress (Tissières et al., 1974; Brown et al., 2014). Subsequently, it was shown that Hsps are evolutionarily conserved in most organisms and are activated by additional, non-thermal stressors, e.g., heavy metals and oxidative stress (Burnie et al., 2006; Soo et al., 2008; Wirk, 2011; Cuéllar-Cruz et al., 2014). Furthermore, many studies have revealed important roles for Hsps in the growth and virulence of *C. albicans* (Leach et al., 2012b; Becherelli et al., 2013; O'meara and Cowen, 2014). Hsps are widely distributed in *C. albicans* and involved in many cellular pathways, such as calcium-calcineurin, MAPK, Ras1-cAMP-PKA, and cell cycle control signaling. Many signaling molecules in these pathways are client proteins of Hsps. Moreover, many studies have demonstrated that Hsps confer *C. albicans* resistance to antifungal drugs by regulating these signaling pathways. Therefore, targeting Hsps pharmacologically or genetically could enhance the sensitivity of *C. albicans* to traditional antifungal drugs and reduce its pathogenicity (Fiori et al., 2012; Mayer et al., 2013; Li and Sun, 2016). Thus, Hsps and other signaling molecules of Hsps-associated pathways are potential novel antifungal targets against candidiasis. Hsp-associated signaling pathways and potential antifungal targets based on these signaling pathways in *C. albicans* are illustrated in Figure 1. The research identifying antifungal agents that target Hsps and Hsp-associated signaling pathways is summarized in Table 1.

**Table 1:** Antifungal agents targeting Hsps and Hsp-associated signaling pathways.

**Antifungal targets**	**Antifungal agents**		**Combined drugs**	***Candida* spps**.	**Methods and results**	**References**
Hsp90	GdA	Hsp90 inhibitors	FLC	*C. albicans* wild-type biofilm	FICI, SY	Robbins et al., 2011
			Micafungin	*C. albicans l*aboratory strains (*n* = 2)	An MIC assay, SY	Singh et al., 2009
	RAD		FLC VOR	Azole-resistant *C. albicans* isolate (T118)	Etest (AB Biodisk),SY	Cowen and Lindquist, 2005
			Micafungin	*C. albicans* laboratory strains (*n* = 2)	An MIC assay, SY	Singh et al., 2009
	17-AAG		FLC	FLC-resistant *C. albicans* isolate (CaCi-2)	Etest (AB Biodisk),SY	Cowen et al., 2009
	17-DMAG		FLC	FLC-resistant *C. albicans* isolate (CaCi-2)	Etest (AB Biodisk),SY	Cowen et al., 2009
	Hsp990		FLC	FLC-resistant *C. albicans* (*n* = 20)	FICI, agar diffusion tests, T-K curves, SY	Li et al., 2015
	Efungumab (Mycograb)	Anti-Hsp90 antibodies	FLC	FLC-resistant *C. albicans* isolate	FICI, IN	Matthews et al., 2003
				FLC-susceptible *C. albicans* isolate	FICI, SY	
			AMB	*C. albicans* isolates (*n* = 2)	FICI, SY	
			Caspofungin	*C. albicans* isolates (*n* = 2)	FICI, SY or IN	Hodgetts et al., 2008
	MycograbC28Y		AMB	*C. albicans* strains (*n* = 2, ATCC 90028 and ATCC 24433)	FICI, SY	Richie et al., 2012
	Trichostatin A	HDAC inhibitors	Miconazole	*C. albicans* wild-type	A rapid selection regimen, SY	Robbins et al., 2012
			FLC ITR Miconazole	*C. albicans* strains (*n* = 8)	Broth microdilution assay, SY or IN	Smith and Edlind, 2002
	MGCD290		Posaconazole FLC VOR	*C. albicans* strains (*n* = 11)	FICI, SY or IN	Pfaller et al., 2009
calcium- calcineurin signaling pathway calcium- calcineurin signaling pathway	FK506	Calcineurin inhibitors	Posaconazole	*C. albicans* (*n* = 10)	FICI, SY	Chen et al., 2013
			FLC VOR ITR	Azole-susceptible *C. albicans* isolates (*n* = 5)	FICI, ΔE method, T-K curves, SY or IN	Sun et al., 2008
			FLC	*C. albicans* wild-type biofilm (*n* = 3)	FICI, SY	Uppuluri et al., 2008
	CsA		FLC	*C. albicans* biofilm (*n* = 4)	FICI, SY or IN	
	Verapamil	CCB	–	*C. albicans* strains (*n* = 4)	Inhibitory effect on hyphae	Yu et al., 2014a
	Amlodipine Nifedipine Benidipine Flunarizine		FLC	FLC-resistant *C. albicans* isolates (*n* = 2)	FICI, T-K curves, SY	Liu et al., 2015
MAPK signaling pathways	Cercosporamide	Pkc1 kinase inhibitors	An echinocandin analog	*C. albicans* strains (*n* = 2)	FICI, SY	Sussman et al., 2004
Ras1-cAMP- PKA signaling pathway	MDL-12330A	AC inhibitors	FLC ITR Terbinafine	*C. albicans* wild-type (SC5314) FLC-resistant *C. albicans* (*n* = 6) FLC-susceptible *C. albicans* (*n* = 6)	Broth microdilution assays, SY	Jain et al., 2003
	Retigeric acid B		–	*C. albicans* strains (*n* = 5)	Broth microdilution assays, MIC_80_ = 8–16 μg/mL	Chang et al., 2012
	Staurosporine	A kinase inhibitor targeting Cyr1 and PKA	FLC Micafungin Llylamine Terbinafine	FLC-resistant *C. albicans* isolate (CaCi-2)	YPD plate assay, SY or IN	Lafayette et al., 2010; Xie et al., 2017

*VOR, Voriconazole; ITR, Itraconazole; MIC, Minimum inhibitory concentrations; T-K curves, time-kill curves; SY, synergy; IN, indifferent; YPD, yeast peptone dextrose*.

In this article, we will review the functions of Hsps in *C. albicans*, the roles of Hsps in various intracellular signaling pathways and potential antifungal targets based on Hsp-associated signaling pathways. Several elements of the Hsp family may represent novel antifungal targets against *C. albicans* infections.

## Functions of Hsps in *C. albicans*

As a class of molecular chaperones, Hsps have multiple broad functions within organisms. Generally, Hsps are classified according to their molecular size (Powers and Workman, 2007). In *C. albicans*, six kinds of Hsps with varying molecular sizes have been identified. Four of them, Hsp104, Hsp90, Hsp70, and Hsp60, are adenosine triphosphate (ATP)-dependent high molecular mass Hsps. The other two, Hsp12 and Hsp21, are ATP-independent low molecular mass Hsps with sizes ranging from 12 to 42 kDa (Jaya et al., 2009). Most of these Hsps play significant roles in the growth and virulence of *C. albicans*.

### Hsp104

As a kind of heat-induced molecular disaggregase, Hsp104 was first discovered in *Saccharomyces cerevisiae (S. cerevisiae)* (Sanchez and Lindquist, 1990; Glover and Lindquist, 1998; Jaya et al., 2009). In *C. albicans*, Hsp104 expression increases after transient exposure of cells to high temperature. Hsp104 is a pro-survival mediator in response to increasing temperature, suggesting an important role for this protein in thermotolerance (Sanchez and Lindquist, 1990). In addition, hyphae formed in biofilms by wild-type and Hsp104-reconstituted strains grow in an intertwined appearance. In contrast, hyphae formed by *hsp104*Δ*/*Δ mutants show structural defects, appearing patchy and loose. These mutants also show attenuated pathogenicity in *Caenorhabditis elegans* infection models. These results demonstrate that Hsp104 is required for efficient biofilm formation and contributes to the virulence of *C. albicans* (Fiori et al., 2012). Hsp104 represents a prospective antifungal target against *C. albicans* because of the absence of a cytosolic Hsp104 equivalent in human.

### Hsp90

Hsp90 augments virulence factors and confers antifungal drug resistance to common pathogenic fungi, *C. albicans, Aspergillus fumigatus*, and *Cryptococcus neoformans* (Cordeiro Rde et al., 2016; Lamoth et al., 2016; Chatterjee and Tatu, 2017). The functions of Hsp90 are modulated by post-transcriptional modifications, mainly including phosphorylation, S-nitrosylation, and acetylation in *yeast*. Soroka et al. determined 10 major phosphorylation sites regulated by the dedicated phosphatase Ppt1 in the middle or the C-terminal domain of *yeast* Hsp90. Phosphorylation allows conformational switching and facilitates communication of remote regions within Hsp90 (Soroka et al., 2012). Besides, a cysteine residue located in the C-terminal domains is proved to be conserved in *yeast* and human Hsp90 family. S-nitrosylation of this residue regulates the functions of Hsp90 fast and efficiently (Martínez-Ruiz et al., 2005; Retzlaff et al., 2009). Moreover, the process of histone acetylation regulated by histone acetyltransferases (HATs) and histone deacetylases (HDACs), also known as lysine deacetylases (KDACs), plays a causative role in regulating gene expression (Trojer et al., 2003). Recent studies showed that Hsp90 acetylation has a profound impact on Hsp90 function. Li et al. verified that the key acetylation sites on *C. albicans* Hsp90 are lysine 30 and 271 and substitutions at these residues phenocopy inhibition of Hsp90 (Li et al., 2017). Furthermore, HDAC inhibitors, such as trichostatin A (TSA) and MGCD290, a Hos2 HDAC inhibitor, have been shown to abrogate Hsp90-dependent azole resistance in *C. albicans*. These results illustrate that acetylation regulates Hsp90 function and then governs antifungal drug resistance with broad therapeutic prospects (Smith and Edlind, 2002; Pfaller et al., 2009; Robbins et al., 2012). The challenge of successfully targeting HDACs in the treatment of candidiasis is to develop stable and specific inhibitors capable of distinguishing pathogens from host.

Hsp90 is one of the most intensely studied Hsps in *C. albicans*. Many studies demonstrate that Hsp90 plays an important role in thermal stability, morphogensis, cell cycle regulation, apoptosis, and drug resistance in *C. albicans* (Leach et al., 2012b; O'meara and Cowen, 2014). During evolution, *C. albicans* retained the conserved protein Hsp90, enabling *C. albicans* to adapt to thermal stress upon colonization of warm-blooded animals or thermally buffered niches (Leach et al., 2012a). Hsp90 governs cellular circuitry required for crucial morphogenetic transitions from yeast to filament (Shapiro et al., 2009, 2012b). Genetically or pharmacologically inhibiting the functions of Hsp90 blocks the growth, maturation, and dispersal of *C. albicans* biofilms *in vitro* (Robbins et al., 2011). In addition, the filaments generated by compromised Hsp90 are similar to those seen during cell cycle arrest. Further studies illustrate that *C. albicans* Hsp90 regulates the progression of yeast-form growth via interaction with diverse control elements of the cell cycle (Berman, 2006; Senn et al., 2012). In addition, Léger et al. demonstrated that in *C. albicans* the metacaspase Mca1p appears to degrade several major Hsps, including Hsp90, which weakens cellular defenses and leads to apoptosis (Léger et al., 2015). Hence, Hsp90 is tightly related by apoptosis, and inhibiting Hsp90 reduces apoptosis in *C. albicans* (Dai et al., 2012). Furthermore, Hsp90 contributes to the appearance and maintenance of antifungal drug resistance in *C. albicans*. Impairing the function of Hsp90 *in vitro* could enhance the efficacy of azoles against *C. albicans* planktonic cells and biofilms (Cowen and Lindquist, 2005; Cowen et al., 2009; Shapiro et al., 2009; Robbins et al., 2011). Hsp90 inhibitors have been shown to have synergistic effects in combination with fluconazole (FLC) against FLC-resistant *C. albicans* (Cowen et al., 2009; Li et al., 2015), so it follows that interfering with the physiological activity of Hsp90 could be a promising strategy to treat candidiasis (Veri and Cowen, 2014).

The currently available agents that pharmacologically target Hsp90 are primarily Hsp90 inhibitors and anti-Hsp90 antibodies. As mentioned above, high-molecular-mass Hsps are ATP-dependent and the N-terminal domain of Hsp90 is an ATP binding site. This domain is highly conserved across species and is essential for the function of Hsp90 (Jackson, 2013). Currently available Hsp90 inhibitors include radicicol (RAD), geldanamycin (GdA), analogs of GdA, including 17-allylamino-17-dimethoxygeldanamycin(17-AAG) and 17-dimethylaminoethylamino-17-demethoxygeldanamycin(17-DMAG) and non-GdA Hsp90 inhibitors, e.g., NVP-HSP990 (HSP990). Each of these binds with the same site in the N-terminus of Hsp90 (Singh et al., 2009; Wirk, 2011; Li et al., 2015). Singh et al. reported that both RAD and GdA reduce the tolerance of *C. albicans* to echinocandins and that each of them exerts synergistic antifungal effects in combination with echinocandins (Singh et al., 2009). Additionally, Cowen et al. found that GdA analogs have synergistic effects in combination with FLC against *C. albicans* (Cowen et al., 2009). However, GdA and its derivatives have not been fully developed for clinical use because of their limited physiochemical properties and severe renal and gastrointestinal cytotoxicity (Kim et al., 2009). This makes non-GdA Hsp90 inhibitors more attractive for therapeutic development. Li et al. demonstrated that HSP990, a non-GdA Hsp90 inhibitor, synergistically combines with FLC against FLC-resistant *C. albicans* and *C. albicans* biofilms both *in vitro* and *in vivo*. HSP990 was also shown to exhibit low cytotoxicity in human umbilical vein endothelial cells (Li et al., 2015). This finding is a promising prospect for the clinical application of non-GdA Hsp90 inhibitors to treat *C. albicans* infections. Although the highly conserved structure of eukaryotic Hsp90 has hindered the development of Hsp90 inhibitors for clinical application, it is promising to find drugs with low cytotoxicity, like HSP990, and there is future potential to find specific targets of *C. albicans* Hsp90.

Another way to inhibit Hsp90 is via stimulation of the host immune response (Bugli et al., 2013). Efungumab (Mycograb), a well-known recombinant monoclonal anti-Hsp90 antibody, binds to the middle domain of Hsp90, effectively inhibiting communication between the terminal regions of Hsp90 and preventing necessary conformational changes (Karwa and Wargo, 2009). The discovery of efungumab against *C. albicans* stemmed from the observation that the presence of anti-Hsp90 antibody is associated with recovery in patients with invasive candidiasis treated with amphotericin B (AMB) (Matthews et al., 1984, 1987). Subsequently, Matthews et al. identified that efungumab exerts antifungal effects alone and synergistically when combined with FLC, AMB, and caspofungin against *C. albicans in vitro* and *in vivo* (Matthews et al., 2003; Hodgetts et al., 2008). Meanwhile, the *in-vivo* pharmacodynamics of efungumab have been confirmed in a murine model. After intravenous administration, it is rapidly distributed into tissues and cleared from the circulation (Matthews et al., 2003; Louie et al., 2011). A blinded and randomized clinical trial further explained the effects of efungumab. The use of efungumab plus lipid-associated AMB produces significant clinical improvement compared to the use of amphotericin B plus placebo for patients with invasive candidiasis (Pachl et al., 2006). In consideration of quality concerns, such as heterogeneity in molecular weight and conformational structure, and safety concerns about adverse effects, such as cytokine release syndrome and hypertension, efungumab was not accredited by the Committee for Medicinal Products for Human Use (CHMP) (Bugli et al., 2013). In response to circumvent these problems for more stability, Louie et al. designed a new compound Mycograb C28Y and identified its synergistic effect in combination with AMB against *C. albicans in vitro*. It is worth noting that Mycograb C28Y lacks efficacy in a murine model with invasive candidiasis (Louie et al., 2011). Besides, the synergistic effect of Mycograb C28Y combined with AMB against *C. albicans* can be reproduced by a wide range of unrelated proteins, indicating that Mycograb C28Y has multiple targets in *C. albicans* (Richie et al., 2012). These findings suggest that complex mechanisms of Mycograb C28Y acting on *C. albicans* need to be further explored. Development of anti-Hsp90 antibodies as novel antifungal agents is a promising therapeutic approach for candidiasis in view of their excellent antifungal effects and clinical efficacy. Certainly, animal experiments and clinical trials are required to confirm the safety and efficacy before anti-Hsp90 antibodies could be contemplated for a clinical use in the future.

### Hsp70

Hsp70 is highly conserved among most species from bacteria to mammals. Hsp70 from different sources has similar biochemical properties because of a high degree of conservation of N-terminal domains; each has a high-affinity ATP-binding site and a peptide-binding site (Craig et al., 1993). Cell surface Ssa1 and Ssa2 are the major members of the Hsp70 family in *C. albicans* (López-Ribot et al., 1996; Eroles et al., 1997). It has been suggested that Ssa1 and Ssa2 exert both positive and negative effects on the growth and virulence of *C. albicans*. On the one hand, Ssa1 and Ssa2 induce host cell endocytosis leading to *C. albicans* mediated pathogenic host cell interactions and increased virulence (Sun et al., 2010). *C. albicans Ssa*Δ*/*Δ mutants exhibit attenuated virulence both *in vitro* and *in vivo*. On the other hand, Ssa1 and Ssa2 are receptors of some antimicrobial peptides that exert antifungal effects. For instance, salivary histatin5 (Hst5) has a high affinity for Ssa proteins on the cell wall of *C. albicans*, facilitating the import of Hst5 (Li et al., 2006; Vylkova et al., 2006, 2007), which is required for Hst5 to exert fungicidal activity against *C. albicans*. Moreover, Maneu et al. discovered that *C. albicans* Ssb1 encoded by *SSB1* has 85% amino acid identity as Ssb1 and Ssb2 of *S. cerevisiae* Hsp70 family (Maneu et al., 1997). Expression of *C. albicans SSB1* complements *S. cerevisiae SSB1 SSB2* double mutant phenotype, indicating that *C. albicans* Ssb1 probably act as a molecular chaperone on the translating ribosomes (Maneu et al., 2000). Msi3, while technically in the Hsp70 family is the homolog of the *S. cerevisiae* Sse1 (human Hsp110). They are regarded as co-chaperones since they are nucleotide exchange factors for Ssa. Msi3 is essential for survival *in vitro* as well as for the establishment of *C. albicans* infections in a mouse model (Nagao et al., 2012). In addition, the mutant strain *tetMsi3*, whose expression of *MSI* is repressed, exhibits higher susceptibility to FLC than does the control strain. Furthermore, *tetMsi3* altered the response to FLC from fungistatic activity to fungicidal activity (Cho et al., 2003; Nagao et al., 2012). Thus, compromising Hsp70 inhibits host cell phagocytosis of *C. albicans* and decreases *C. albicans* antifungal drug resistance. We believe that Hsp70 would also be a potential antifungal target against candidiasis if it conquers the obstacles of safety caused by highly conserved structure of Hsp70 among eukaryotes.

### Hsp60

As immunodominant antigens in humoral and cellular responses, fungal Hsp60 facilitates powerful immunological reactions (Habich et al., 2006). Cross-reactivity between fungal and human Hsp60 may illustrate a potential link between infection and autoimmunity (47). Expression of *C. albicans* Hsp60 mRNA increase upon elevated incubation temperature beyond 35°C (Raggam et al., 2011). Many studies showed that Hsp60 acts as an immunogenic trigger in orchestrating *C. albicans*-related diseases under thermal stress. These findings may contribute to a deeper understanding of host–pathogen relationships (Rajaiah and Moudgil, 2009; Raggam et al., 2011).

### Small Hsps

Unlike the highly conserved sequences among species of high-molecular-mass Hsps, small Hsps share only a short fragment called the acystall within a conserved sequence of the C-terminus. *C. albicans* Hsp12 is a small Hsp primarily expressed under different types of stress, like osmotic and thermal stress (Enjalbert et al., 2003; Smith et al., 2004). Enhanced expression of Hsp12 significantly promotes cell adhesion and germination of *C. albicans*, while decreasing the susceptibility of *C. albicans* to the quorum sensing molecule, farnesol (Davis-Hanna et al., 2008; Fu et al., 2012). In addition, *C. albicans* strains overexpressing *HSP12* are sensitive to itraconazole, ketoconazole, and FLC (Fu et al., 2012). Thus, targeted up regulation of Hsp12 expression is a potential antifungal treatment against *C. albicans*.

Hsp21 is another small Hsp crucial for *C. albicans* to resist specific stressors, including thermal and oxidative stress. Hsp21 is also involved in regulation of glycerol, glycogen, and trehalose homeostasis in response to elevated temperature (Mayer et al., 2012). Additionally, Hsp21 was found to promote the virulence of *C. albicans. Hsp21*Δ*/*Δ mutants form significantly shorter hyphae and exhibit defects in invasive growth (Mayer et al., 2012). Furthermore, *hsp21*Δ*/*Δ *C. albicans* mutants are highly susceptive to a broad range of antifungal drugs (Mayer et al., 2013). Therefore, targeting Hsp21 is a possible treatment strategy for *C. albicans* infection.

## Potential antifungal targets within Hsps-associated intracellular signaling pathways in *C. albicans*

Hsps are involved in a wide variety of intracellular signaling pathways in *C. albicans*. In response to activation of diverse signaling pathways, the heat shock transcription factor (Hsf1) is phosphorylated, resulting in the induction of target *HSP* gene expression via the heat shock element (HSE) (Nicholls et al., 2009). Thus, therapeutic targeting of Hsps could exert antifungal effects or reverse drug tolerance of *C. albicans* to antifungal drugs by disrupting Hsp-related signaling pathways.

The number of available Hsp inhibitors is limited; however, drugs targeting other elements of Hsp-related signaling pathways have been shown to exert antifungal effects. In this section, we will review the Hsp-associated signaling pathways in *C. albicans* and summarize potential antifungal targets in these pathways.

### Calcium-calcineurin signaling pathway

Calcineurin is a conserved calmodulin-dependent phosphatase in pathogenic fungi. Calcineurin is activated by the second messenger calcium and regulates stress responses in fungi (Kraus and Heitman, 2003). Calcineurin is necessary for *C. albicans* to survive during cell membrane stress, cation stress, alkaline pH, and endoplasmic reticulum stress (Cruz et al., 2002; Steinbach et al., 2007). Elements of the calcium-calcineurin signaling pathway, such as various channels, transporters and other proteins or enzymes, are intimately connected to various physiological processes in *C. albicans* (Liu et al., 2015; Li and Sun, 2016).

Singh et al. revealed that impairing the calcineurin function phenocopies inhibition of Hsp90 (Singh et al., 2009), both of which result in decreased drug resistance of *C. albicans* to azoles. Calcineurin is a heterodimer composed of a catalytic subunit (either Cna1 or Cna2) and an activating regulatory subunit (Cnb1) (Cowen and Lindquist, 2005). It is widely accepted that calcineurin is a client protein of Hsp90, which binds to the catalytic subunit of calcineurin in *C. albicans* (Cowen, 2009; Li and Sun, 2016). In addition, downstream targets of calcineurin include the transcription factors Hph1, Hph2, and Crz1, which were initially discovered in *S. cerevisiae* as targets related to stress adaption and drug resistance (Heath et al., 2004; Karababa et al., 2006). Both Crz1 and Hph1 modulate Hsp90-dependent drug resistance (Cowen et al., 2006), and the key mediator of Hsp90-dependent drug resistance is thought to be calcineurin. Furthermore, Hsp90 also regulates apoptosis in *C. albicans* via the calcium-calcineurin signaling pathway (Phillips et al., 2003; Lu et al., 2011). Apoptosis is induced by various environmental stimuli, including hydrogen peroxide (H_2_O_2_), acetic acid (AA) as well as by drug treatment with AMB in *C. albicans*. Caspase, encoded by the gene *CaMCA1*, is an essential enzyme involved in apoptosis. Dai et al. demonstrated that compromising Hsp90 inhibits *CaMCA1* expression, decreasing caspase activity upon exposure to apoptotic stimuli. Thus, compromised Hsp90 reduces *C. albicans* apoptosis, partially via down regulation of the calcineurin-caspase signaling pathway (Dai et al., 2012). In conclusion, calcineurin interacts with Hsp90, and the activated calcineurin-Hsp90 complex regulates the stress response, drug resistance, and apoptosis in *C. albicans* (Juvvadi et al., 2014).

Hsp70 participates in the calcium-calcineurin signaling pathway as well. Msi3, a member of the Hsp70 family, binds Cgr1 during high levels of expression at an early stage of the yeast–hypha transition (Cho et al., 2001, 2003). The relationship between Msi3 and Cgr1 signifies a functional role of Hsp70 in addition to its role in thermal adaptation (Cho et al., 2003). Furthermore, *MSI3* and certain calcineurin-dependent genes are highly expressed in response to FLC treatment. Increased Msi3 leads to expression of the calcineurin dependent genes *UTR2* and *PLC3* in the wide-type strain. However, in the mutant strain *tetMsi3*, up-regulation of *MSI3* is lost in response to FLC treatment, and the expression of calcineurin-dependent genes remains stable. Therefore, induction of calcineurin-dependent gene expression is required for up-regulation of *MSI3* expression. These findings illustrate that Msi3 confers FLC resistance in part by activating the calcium-calcineurin signaling pathway. Nagao et al. speculated that Msi3 functions cooperatively with Hsp90 as a cochaperone or through an as yet undiscovered mechanism to activate the calcineurin signaling pathway (Nagao et al., 2012). Thus, the mechanism for Hsp70 regulation of the calcineurin signaling pathway in *C. albicans* requires further elucidation.

Hsp90 and Hsp70 both participate in the calcium-calcineurin signaling pathway and are crucial to *C. albicans*' drug resistance. Agents targeting calcium, the trigger of Hsp-related calcium-calcineurin signaling show antifungal effects. Inhibitors of calcineurin, cyclosporine A (CsA), and tacrolimus (FK506) inhibit calcineurin by distinct mechanisms (Hemenway and Heitman, 1999). CsA binds with Cpr1, a peptidyl prolyl cis-trans isomerase (cyclophilin A) to calcineurin function. FK506 binds to the structurally unrelated peptidyl-prolyl cis-trans isomerase FKBP12 to block function of calcineurin. Both CsA and FK506 have potent synergistic effects in combination with azoles against *C. albicans* both *in vitro* and *in vivo* (Sun et al., 2008; Uppuluri et al., 2008; Chen et al., 2013). Juvvadi et al. identified a novel serine-proline rich region that is unique to filamentous fungi and absolutely absent in human calcineurin. It has been shown that phosphorylation of the serine-proline rich region regulates the functions of calcineurin in *A. fumigatus* (Juvvadi et al., 2013). Thus, it is prospective to realize the possibility of inhibiting fungal calcineurin specifically as an antifungal strategy. Additionally, calcium channel blockers (CCB) that disturb cellular calcium homeostasis in *C. albicans* have synergistic antifungal effects in combination with FLC (Yu et al., 2014a,b; Liu et al., 2016). Yu et al. verified that verapamil inhibits hyphal development and gastrointestinal colonization of *C. albicans* (Yu et al., 2014a). Furthermore, Liu et al. identified four CCBs, amlodipine, nifedipine, benidipine, and flunarizine, which have synergistic effects with FLC against resistant *C. albicans* strains (Liu et al., 2016). It may provide a potential therapy to combine CCBs with azoles against *C. albicans* infections in clinic. These discoveries indicate that targeting components of the Hsp-associated calcium-calcineurin signaling pathway could exert antifungal characteristics and/or reverse drug tolerance in *C. albicans*.

### MAPK signaling pathways

Mitogen activated protein (MAP) kinase signaling pathways are conserved in eukaryotic cells and regulate growth and adaptations stress, including thermal stress, apoptosis, and inflammation. This pathway includes three types of kinases: MAP kinase kinase kinase (MAPKKK), MAP kinase kinase (MAPKK), and MAP kinase (MAPK). When upstream signals act on MAPKKK, MAPKKK is phosphorylated and in turn phosphorylates MAPKK, which in turn phosphorylates MAPK. Ultimately, MAPK signals to downstream transcription factors in order to develop adaptive responses. Thus, MAPK signaling pathways are activated by many extracellular stimuli and mediate signal transduction from the cell surface to the nucleus (Monge et al., 2006). Four MAPK signaling pathways have been identified in *C. albicans*: the Mkc1 pathway, related to cell wall integrity (Navarro-García et al., 1995); the Hog1 pathway, mainly participating in osmotic, oxidative, and other stress adaptions (Smith et al., 2004; Correia et al., 2016); the Cek1 pathway, involved in mating and starvation (Csank et al., 1998; Chen et al., 2002); and the Cek2 pathway, crucial for mating (Chen et al., 2000, 2002). Each of these MAPK signaling pathways plays important roles in the growth and virulence of *C. albicans*.

MAPK signaling pathways are intimately associated with heat shock responses in *C. albicans* (Brown et al., 2014). Leach et al. illustrated that *C. albicans* Hsp90 modulates the activity of Hsf1 in response to thermal stress over the short term. In addition, the components of MAPK-Cek1, Hog1, and Mkc1-are all client proteins of Hsp90. Compromising Hsp90 function inhibits cell wall biogenesis in *C. albicans* via impairing activation of Cek1, Hog1, and Mkc1. Thus, Hsp90 modulates long-term thermal adaption via Mkc1-, Hog1-, and Cek1- mediated cell wall remodeling (Leach et al., 2012a; Ene et al., 2015).

Further studies have confirmed that Hsp-associated MAPK signaling pathways contribute to the virulence of *C. albicans* and confer drug tolerance. The Mkc1 MAPK pathway is composed of the MAPKKK Bck1, the MAPKK Mkk1 and the terminal transcription factors Swi4/Swi6 (Lafayette et al., 2010; Román et al., 2015). Mediated by protein kinase C (Pkc1), this pathway is responsible for maintaining the integrity of the cell wall during growth, morphogenesis, and cell wall stress in *C. albicans* (Navarro-García et al., 1995; Lafayette et al., 2010). The cell wall is the first point of contact for antifungal drugs and represents an attractive therapeutic target in fungal pathogens (Cullen and Edgerton, 2016). As mentioned above, Hsp90 interacts with Mkc1, and Hsp90 and Hsp70 help to maintain phosphorylation of activated Pkc1 and control the degradation of fully primed and activated Pkc1 (Lum et al., 2013). Therefore, inhibiting Hsp90 both *in vitro* and *in vivo* decreases drug resistance to ergosterol biosynthesis inhibitors via destabilization of the terminal MAPK, Mkc1 of Pkc1-Mkc1 signaling pathway in *C. albicans* (Singh et al., 2009; Lafayette et al., 2010). Furthermore, LaFayette et al. speculated that the Pkc1-Mkc1 and calcineurin pathways, controlled by Hsp90 independently, may regulate drug resistance of *C. albicans* to ergosterol biosynthesis inhibitors through a single target (Lafayette et al., 2010). In addition, as a member of the Hsp70 family, *C. albicans* Ssa1 and Ssa2 act as invasins. Ssa1 and Ssa2 combine with host cell cadherins and facilitate host cell endocytosis, allowing *C. albicans* to invade host cells (Sun et al., 2010). Saraswat et al. suggested that Ssa1 binding with mucin Msb2 detects and regulates thermal stress adaptations, such as survival and hyphae formation at high temperature through the Pkc1-Cek1 signaling pathway (Saraswat et al., 2016). *Ssa1*Δ*/*Δ and *Msb2*Δ*/*Δ mutants were shown to exhibit defective phosphorylation of Mkc1 (P~Mkc1) and P~Cek1, especially at high temperatures. Moreover, some small Hsps are also involved in MAPK signaling. Genetic deletion of *HSP21* prevents phosphorylation of Cek1 at elevated temperatures, indicating that phosphorylation of Cek1 during thermal stress is Hsp21-dependent (Mayer et al., 2012). In addition, expressions of *C. albicans HSP12* is regulated by the Hog1 stress response. It has been shown that devitalized Hog1 represses *HSP12* expression. However, stress mediated by activated Hog1 abolishes this repression (Smith et al., 2004; Fu et al., 2012).

These results all verify that Hsps govern the MAPK signaling pathways that play significant roles in the growth and virulence of *C. albicans*. As a result, inhibiting Hsps or other elements of MAPK signaling should disrupt growth and reduce virulence of *C. albicans*. Sussman et al. found that cercosporamide, a selective and potent fungal Pkc1 kinase inhibitor, acts as a broad-spectrum natural antifungal compound against *C. albicans*. Furthermore, cercosporamide, in combination with an echinocandin analog, exerts synergistic antifungal effects against *C. albicans* (Sussman et al., 2004). This finding suggests a potential efficient combination strategy to treat *C. albicans* infections. Hopefully, more drugs targeting molecules of the MAPK signaling pathways will be discovered and may help to improve the treatment of *C. albicans* infections.

### RAS1-CAMP-PKA signaling pathway

As a conserved small GTPase on the plasma membrane of eukaryotic cells, Ras regulates a key cyclic Adenosine monophosphate (cAMP)-dependent protein kinase A (PKA) pathway (Mösch et al., 1999; Leberer et al., 2001; Piispanen et al., 2011). In *C. albicans*, the Ras1-cAMP-PKA signaling pathway is primarily composed of the proteinRas1, adenylate cyclase (AC), cAMP, PKA, and its downstream targets (Hogan and Sundstrom, 2009). Ras1 is described as a “molecular switch,” bound to either guanosine triphosphate (GTP) during an activated state by interaction with guanosine nucleotide exchange factors (GEFs) or to guanosine diphosphate (GDP) during inactivated states through interaction with GTPase activator proteins (GAPs) (Fortwendel, 2012). Upon Ras1 activation, Ras1-GTP binds to AC and catalyzes production of the second messenger cAMP. Subsequently, the increase of intracellular cAMP activates cAMP-dependent PKA that phosphorylates a series of transcription factors that control phenotype, metabolism, stress adaption, proliferation, and other functions (Broach, 1991; Rolland et al., 2002; Cassola et al., 2004; Li and Wang, 2013). Further studies show that Efg1, a transcription factor, in parallel with other unidentified signaling molecules, is the terminal point of the Ras1-cAMP-PKA pathway in *C. albicans* (Bockmühl and Ernst, 2001; Shapiro et al., 2009). Moreover, the Ras1-cAMP-PKA signaling pathway is also involved in normal physiological activities, e.g., acceleration of apoptosis (Phillips et al., 2006), as well as several pathogenic behaviors, such as cell adhesion, hyphal morphogenesis, biofilm formation, and white-to-opaque switching (Inglis and Sherlock, 2013).

Studies have conclusively demonstrated that *C. albicans* Hsps control growth and virulence of *C. albicans* via interaction with the Ras1-cAMP-PKA pathway (Leberer et al., 2001; Shapiro et al., 2009). Compromising Hsp90 function induces a transition from yeast to hypha growth, while attenuating virulence, in a murine model of *C. albicans* infection. These phenotypes are likely due to *C. albicans*' requirement for morphogenetic flexibility to attain virulence (Shapiro et al., 2009, 2012a). Elevated temperature beyond 37°C impairs Hsp90 activity and reverses Hsp90-mediated repression of morphogenetic progress (Shapiro et al., 2012b; Mayer et al., 2013). Shapiro et al. discovered that a *C. albicans* strain with an active *RAS1* allele (*Ras1*^*V13*^) and a *C. albicans* strain heterozygous for *HSP90* remain as yeast. In contrast, the combination of *Ras1*^*V13*^ and reduced levels of Hsp90 leads to filamentation. Furthermore, deletion of one *IRA2* allele has no effect on Ras1 activity in a strain containing wild type Hsp90; however, deleting one *HSP90* allele in the *IRA2*Δ*/*Δ heterozygote enhances filamentation. In conclusion, genetic epistasis analyses suggest that Hsp90 promotes *C. albicans* morphogenesis by repressing Ras1-cAMP-PKA signaling in a temperature-dependent manner (Shapiro et al., 2009). Other studies have focused on specific interactions between Hsp90 and the Ras1-cAMP-PKA signaling pathway in *C. albicans*. Shapiro et al. demonstrated that genetic depletion of *SGT1* phenocopies compromise of Hsp90 function. They described Sgt1 as a co-chaperone that physically interacts with Hsp90. Moreover, they asserted that Cyr1, the AC of the Ras1-cAMP-PKA signaling pathway, interact with Sgt1 and Hsp90, thereby controlling Ras1-cAMP-PKA signaling (Shapiro et al., 2009, 2012b). We know that tight control of trehalose, regulated by the G protein-coupled receptor (Gpr1), is required for *C. albicans* morphogenesis. Suggesting that trehalose is a possible link between the Ras1-cAMP-PKA pathway and the Hsp90-mediated regulation of morphogenesis (Serneels et al., 2012). In summary, Hsp90 regulates *C. albicans* morphogenesis and pathogenesis in response to environmental stress via the Ras1-cAMP-PKA signaling pathway. Dodecanol-and farnesol-induced expression of *HSP12* stimulates budding growth in wild type *C. albicans* and is dependent on Ras1-cAMP-PKA signaling (Davis-Hanna et al., 2008; Fu et al., 2012). What's more, Hsp21, which potentiates resistance of *C. albicans* to commonly used antifungal drugs, also lies downstream of the Ras1-cAMP-PKA signaling pathway (Harcus et al., 2004; Mayer et al., 2013).

In conclusion, Hsps regulate the virulence properties of *C. albicans*, especially hyphal morphogenesis, via Ras1-cAMP-PKA signaling. Targeting Hsps-related Ras1-cAMP-PKA signaling is also a potential antifungal strategy against *C. albicans*. Jain et al. have shown that MDL-12330A, an inhibitor of mammalian AC, has a synergistic effect in combination with azoles against both azole-susceptible and-resistant strains of *C. albicans* (Jain et al., 2003). In addition, Xie et al. confirmed that staurosporine, a protein kinase inhibitor, abrogates fungal drug resistance via targeting AC and the cAMP-dependent PKA (Xie et al., 2017). The lichen-derived small molecule retigeric acid B that inhibits AC activity significantly inhibits the filamentation of *C. albicans*. Moreover, it attenuates the virulence of *C. albicans*, leading to prolonged survival in nematodes models (Chang et al., 2012). In short, drugs targeting the Ras1-cAMP-PKA signaling pathway and mechanisms for these drugs need to be further studied for treatment of candidiasis.

### Cell cycle control pathways

In fungi, extracellular and intracellular signals govern the cell cycle, controlling cell division and differentiation. Fungal cells exhibit different morphologies, affected by the activity of cyclin-dependent kinases. Cell cycle regulation is pivotal for the virulence and infectious development of fungal pathogens (Berman, 2006; Pérez-Martín et al., 2016).

Leach et al. observed that Hsf1 and Hsp90 orchestrate temperature-dependent global transcriptional remodeling of chromatin architecture in *C. albicans*, indicating Hsps may participate in movement of the cell nucleus (Leach et al., 2016). Further studies suggest that Hsps are involved in the cell cycle control pathways of *C. albicans*. Senn et al. found that filaments of *C. albicans* induced by compromised Hsp90 are neither pseudohyphae nor hyphae-like, but rather are similar to filaments induced by cell cycle arrest (Senn et al., 2012). This finding implies that Hsp90 is involved in cell cycle control and affects morphogenesis of *C. albicans*. Further studies illustrate that Cdc28, a kind of cyclin-dependent kinase in *C. albicans*, is normally stable, but its depletion leads to filamentous growth (Umeyama et al., 2006). However, levels of Cdc28 are markedly reduced in response to Hsp90 depletion, while *CDC28* transcript levels remain stable. Therefore, reduced levels of Hsp90 may result in instability of Cdc28. Thus, Senn et al. speculated that Cdc28 is a candidate for an Hsp90 client protein and is one of the targets of Hsp90's influence on cell cycle control in *C. albicans* (Senn et al., 2012). Additionally, Hsp90 is required for the stability of two *C. albicans* mitotic cyclins, Clb2, and Clb4 (Bensen et al., 2005). Nevertheless, *CLB* deletion does not lead to Hsp90 inhibition, and so it is less likely to contribute to Hsp90's impacts on morphogenesis (Bensen et al., 2005; Senn et al., 2012). Moreover, Shapiro et al. showed that Hms1, a transcriptional regulator, contributes to morphogenesis induced by elevated temperature or impaired Hsp90 in *C. albicans*. Hms1 lies downstream of the cyclin Pcl1 and the cyclin-dependent kinase Pho85. In response to inhibition of Hsp90, Hms1 promotes filamentous growth by activating the expression of *UME6* and *RBT5*. Meanwhile, deleting *HMS1* improves the survival rates of *C. albicans* infection in metazoan models (Shapiro et al., 2012a). These data indicate a potential antifungal strategy for treating *C. albicans* infections by targeting elements in the Pho85-Pcl1-Hms1 pathway. These findings demonstrate that Hsp90 governs morphogenesis via multiple elements of cell cycle control pathways in *C. albicans*. Thus, modulation of Hsp90 is a promising antifungal strategy to treat *C. albicans* infections.

### Others

Other signaling pathways in *C. albicans* have been shown to be regulated by Hsps. Leach et al. first revealed a molecular link between membrane fluidity and the heat shock response (Leach and Cowen, 2014). In response to elevated temperature, the E3 ubiquitin ligase Rsp5, which controls expression of *OLE1* via transcription factor Spt23, is a hypothesized early sensor of temperature. The *OLE1* gene encodes a fatty acid desaturase, and decrease of *OLE1* triggers expression of *FAS2*, which encodes a fatty acid synthase. Moreover, the decrease of *OLE1* prevents activation of Hsf1 and reduces expression of *HSP* in response to heat shock. This finding confirms the link between membrane fluidity and the heat shock response. In addition, *OLE1* not only controls levels of fatty acid desaturase, which regulates fluidity of *C. albicans* membrane, but also facilitates hyphae development, a crucial process for *C. albicans* virulence (Krishnamurthy et al., 2004; Noverr and Huffnagle, 2004). Thus, disrupting Hsp-regulated membrane fluidity is a potential antifungal strategy against *C. albicans*.

Robbins et al. reported that impairing Hsp90 governs dispersion and reverses antifungal drug tolerance of *C. albicans* biofilms via down-regulation of matrix glucan (Robbins et al., 2011). They illustrated that Hsp90 regulates glucan levels either by affecting Fks1, an important synthase for the production of matrix glucan, or by affecting Zap1 and its downstream targets Gca1 and Gca2, controlling the hydrolytic release of β-glucan fragments to the matrix. Existence of matrix glucan is a barrier for drugs in reaching *C. albicans* and promotes its invasive ability. Therefore, targeting matrix glucan via Hsp90 disruption may also be a good therapeutic strategy against *C. albicans* biofilms.

## Conclusion

Initially, Hsps in *C. albicans* were considered simply a series of proteins generated in response to thermal stress. Further studies suggest that in addition to regulating thermal adaptations, Hsps act as molecular chaperones that interact with many molecules in diverse signaling pathways, such as the calcium-calcineurin signaling pathway, MAPK signaling pathways, the Ras1-cAMP-PKA signaling pathway and cell cycle control pathways. These Hsp-associated pathways are essential for controlling basic physiological activities and virulence of *C. albicans*. Further studies have shown that agents targeting Hsps and elements of Hsp-associated pathways exert antifungal effects and/or reverse tolerance of *C. albicans* to traditional antifungal drugs. These findings support the hypothesis that understanding the link between Hsps and its many signaling partners could lead to the elucidation of several novel antifungal targets to be explored in *C. albicans*.

## Author contributions

YG wrote the review and created Figure 1 and Table 1, SS, CY, and TL helped with it.

### Conflict of interest statement

The authors declare that the research was conducted in the absence of any commercial or financial relationships that could be construed as a potential conflict of interest.
